# Phylogenetic Reconstruction of the *Legionella pneumophila* Philadelphia-1 Laboratory Strains through Comparative Genomics

**DOI:** 10.1371/journal.pone.0064129

**Published:** 2013-05-22

**Authors:** Chitong Rao, Hadas Benhabib, Alexander W. Ensminger

**Affiliations:** 1 Department of Molecular Genetics, University of Toronto, Toronto, Ontario, Canada; 2 Public Health Ontario, Toronto, Ontario, Canada; University of São Paulo, Brazil

## Abstract

Over 20 years ago, two groups independently domesticated *Legionella pneumophila* from a clinical isolate of bacteria collected during the first recognized outbreak of Legionnaires’ disease (at the 1976 American Legion’s convention in Philadelphia). These two laboratory strains, JR32 and Lp01, along with their derivatives, have been disseminated to a number of laboratories around the world and form the cornerstone of much of the research conducted on this important pathogen to date. Nevertheless, no exhaustive examination of the genetic distance between these strains and their clinical progenitor has been performed thus far. Such information is of paramount importance for making sense of several phenotypic differences observed between these strains. As environmental replication of *L. pneumophila* is thought to exclusively occur within natural protozoan hosts, retrospective analysis of the domestication and axenic culture of the Philadelphia-1 progenitor strain by two independent groups also provides an excellent opportunity to uncover evidence of adaptation to the laboratory environment. To reconstruct the phylogenetic relationships between the common laboratory strains of *L. pneumophila* Philadelphia-1 and their clinical ancestor, we performed whole-genome Illumina resequencing of the two founders of each laboratory lineage: JR32 and Lp01. As expected from earlier, targeted studies, Lp01 and JR32 contain large deletions in the *lvh* and *tra* regions, respectively. By sequencing additional strains derived from Lp01 (Lp02 and Lp03), we retraced the phylogeny of these strains relative to their reported ancestor, thereby reconstructing the evolutionary dynamics of each laboratory lineage from genomic data.

## Introduction

Beginning in 1976 with a large outbreak of a mysterious flu-like illness at a convention of the American Legion in Philadelphia, an ever-growing community of clinical and research scientists have dedicated themselves to understanding the pathogen behind this illness and the mechanisms by which it causes Legionnaires' disease [1]. *Legionella pneumophila*, the causative agent of Legionnaires' disease, is a gram-negative, facultative intracellular parasite of freshwater protists and an accidental pathogen of humans upon inhalation of contaminated water [2]. *L. pneumophila* persists in environmental reservoirs as an intracellular pathogen of diverse protozoan hosts [3]–[5]. From the perspective of the pathogen, replication in mammalian cells is likely an evolutionary dead end, with no observed transmission between humans even in very large outbreaks of disease [6]. Using an experimental evolution approach, we previously showed that extended *L. pneumophila* propagation in macrophages rapidly selects for several parallel mutations that restrict host range and improves bacterial replication in this novel adaptive environment [7]. Extracellular propagation of these bacteria in the laboratory is also a novel, suboptimal environment for *L. pneumophila* replication, suggesting that the analysis of domesticated genomes might also provide unique insight into the bacterial response to environmental change.

**Figure 1:**
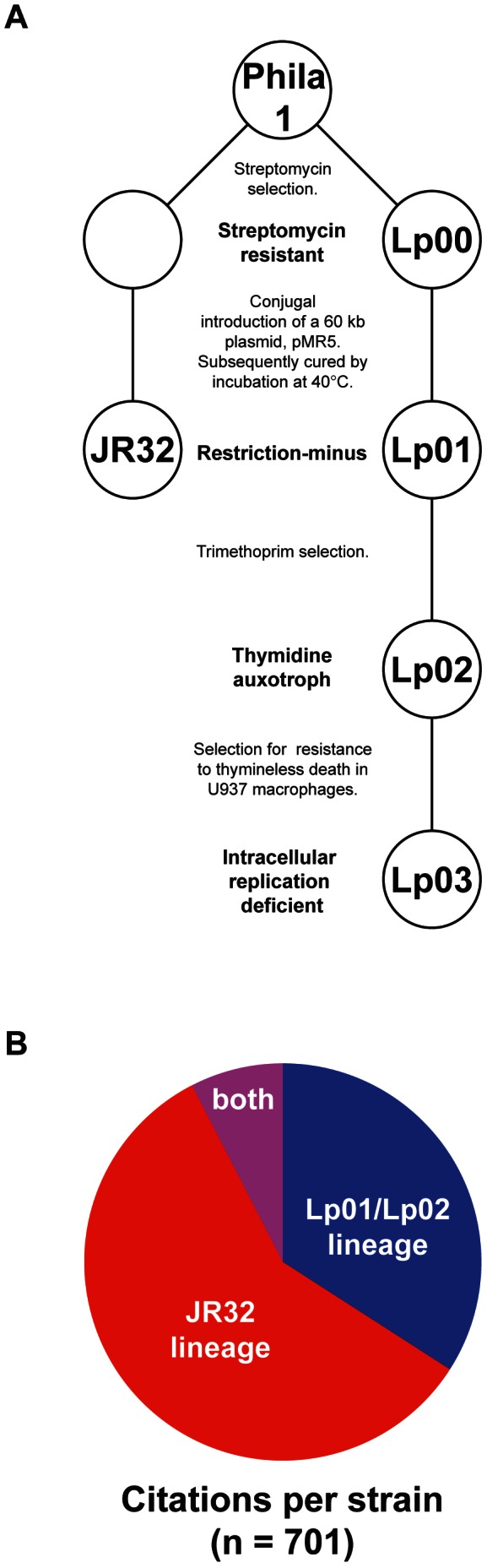
*Legionella pneumophila* Philadelphia-1 laboratory strains. (A) Presumed phylogeny of the laboratory strains of *L. pneumophila* Philadelphia-1 based on the parallel experimental methods used to generate each strain. Both the Lp01 and JR32 lineages were derived from a clinical isolate, *L. pneumophila* Philadelphia-1, collected during the first recognized outbreak of Legionnaires' disease at a 1976 convention of the American Legion in Philadelphia, Pennsylvania. Lp01 was subsequently used to derive a spontaneous thymidine auxotroph Lp02. An avirulent *dotA* mutant, Lp03, reportedly derived from Lp02, is commonly used in studies as a translocation-deficient, intracellular replication defective control. (**B**) Out of a total of 700 publications, an estimate of the relative number which reference the Lp01/Lp02 lineage, JR32, or both. (See Materials and Methods).

In order to molecularly and genetically characterize *L. pneumophila* str. Philadelphia-1, two groups sought to develop laboratory models of the bacterium from clinical isolates collected during the original 1976 outbreak [8], [9]. Initial observations were that undomesticated Philadelphia-1 was a poor recipient in mating with *E. coli* [8] and attempts were made to generate strains more amenable to genetic manipulation. In each laboratory, the first step in domestication was the selection for spontaneous streptomycin resistance on solid media (**fig. 1A** **, step 1**) [8], [9]. Bacterial conjugation with *E. coli* was then used to introduce pMR5, an unstable, 60 kb plasmid [10], [11] into *L. pneumophila*. Streptomycin was used to select against the *E. coli* donor strains (**fig. 1A** **, step 2**) and kanamycin was used to select for transformants that presumably carried one or more spontaneous mutations allowing for increased efficiency to receive and maintain foreign DNA. Each plasmid was subsequently cured by growth at elevated (40°C) temperatures, resulting in two highly tractable model strains, JR32 and Lp01. Despite being separated by a few described phenotypic differences [12], these two strains have provided a foundation for the majority of *L. pneumophila* molecular research over the last 20 years **(** **fig. 1B** **)**.

Whole-genome sequencing (WGS) is a powerful emerging tool for tracing the evolutionary trajectories of pathogens during outbreaks of disease. The identification of single nucleotide polymorphisms (SNPs) can be used to distinguish isolates that previously would have been characterized as identical by other, lower resolution means, such as multilocus sequence typing (MLST) [13]. The power of the unprecedented resolution afforded by WGS has been used to follow the transmission of methicillin-resistant *Staphylococcus aureus* (MRSA) through a hospital ward [14], identify putative “super spreaders” of *Mycobacterium tuberculosis* [15], and measure the evolution of *Burkholderia dolosa* and *Pseudomonas aeruginosa* during colonization of cystic fibrosis patients [16], [17]. Described here, we used similar approaches to follow the presumably simpler evolutionary trajectory of *L. pneumophila* Philadelphia-1 during its domestication and incidental laboratory passage.

## Results

### Whole-genome Resequencing of *L. pneumophila* Philadelphia-1 and Two Independently Domesticated Laboratory Lineages

To identify genetic mutations that may have been selected for during the generation of each of two commonly cited *L. pneumophila* str. Philadelphia laboratory strains (**fig. 1A**), genomic libraries of each were sequenced using the Illumina platform. Paired-end sequence reads from Lp01 and JR32 were reference aligned to the published *L. pneumophila* str. Philadelphia-1 clinical genome [18], the strain background from which they were presumably derived (GenBank accession AE017354.1, see Materials and Methods). To differentiate between true mutations that may have arisen during or after the domestication of each strain and any potential discrepancies between the published genome and the Philadelphia-1 progenitor strain used to generate the Lp01 lineage, we next Illumina sequenced undomesticated Philadelphia-1 for comparison. Very few polymorphisms were detected between this strain and the published sequence. High confidence polymorphisms that reside in regions where reads could be uniquely aligned are listed in **table 1** **.** Notably, one observed polymorphism in *lpg0644* would restore an intact open reading frame (ORF) extending from *lpg0644* through *lpg0645* with homology to the full-length *rtxA* gene that has been reported as both present [19] and absent [20] in the Philadelphia-1 genome (**table 1** **)**. These interpretations, however, are complicated by the tandem repeats found at the *rtxA* locus [21], which poses technical challenges for both Sanger and Illumina sequencing of the region. Repetitive sequence between *lpg0745* and *lpg0746* also makes Illumina sequencing and interpretation difficult. Other deviations from the published sequence were identified in less repetitive regions: *lpg0748*, *lpg0775*, and *lpg2721*. A frameshift at the start of the previously annotated *lpg0775* changes the reading frame such that 73 N-terminal amino acids are included at the start of the ORF. Providing confidence to this prediction, the resulting protein is 100% identical to an *L. pneumophila* str. Paris ortholog, *lpp0840*. Each of these polymorphisms between Philadelphia-1 and the published genome are also present in the two domestic lineages, consistent with their derivation from this progenitor. Several additional mutations identified as unique to each of the domesticated strains, JR32 and Lp01, are listed in **table 2** and **table 3**, respectively**.**

**Table 1:** Polymorphisms between the Philadelphia-1 progenitor and the original published sequence.

Annotated ID	Gene	Base Change^1^	Amino Acid Change
*lpg0644*	N-terminus of *rtxA*	G 688,274 A	Synonymous
*lpg0644*	N-terminus of *rtxA*	C 688,309 del	Frameshift restores full-length *rtxA* gene and merges *lpg0644* and *lpg0645*.
*lpg0745-lpg0746*	“intergenic”	T 816,495 AT 816,562 G	None. Nucleotide substitutions upstream of previously undescribed open reading frame with 100% homology to ortholog *lpp0811*.
*lpg0748*	LPS biosynthesis protein, PseA-like	GT 820,457–820,458 CG	V 368 R
*lpg0775*	glycosyl transferase	G 849,248 del	Changes frame to restore 73 residues to the N-terminus of the protein. CTG start site, 100% homology to ortholog *lpp0840*.
*lpg1228*	hypothetical protein	N 1,355,972 T	No change.
*lpg2721*	glutamine amidotransferase	G 3,073,982 del	Truncates open reading frame by 2 amino acids at the N-terminus. Open reading frame starts at previously annotated M 3.

1 Nucleotide positions within the published *L. pneumophila* Philadelphia-1 genome [18] (GenBank accession AE017354.1).

**Table 2:** JR32 polymorphisms relative to the Philadelphia-1 progenitor.

Annotated ID	Gene	Base Change^1^	Amino Acid Change
*lpg0324*	*rpsL*	A 378,931 G	K 88 R
*lpg0568-lpg0569*	intergenic	AGAT 608,162–608,165 GAAG	None.
*lpg1237*	type II restriction enzyme(Eco47II, Sau 96I)	C 1,366,637 T	G 90 E
*lpg1291*	*qseC (pmrB)*	G 1,419,205 A	T 296 I
*lpg2057-lpg2115*	*tra* region	2,296,869–2,361,074 del	Not applicable
*lpg2180*	*arcB*	G 2,453,675 T	L 653 F
*lpg2372*	hypothetical protein	C 2,677,696 T	S 168 F

1 Nucleotide positions within the published *L. pneumophila* Philadelphia-1 genome [18] (GenBank accession AE017354.1).

**Table 3:** Lp01 polymorphisms relative to the Philadelphia-1 progenitor.

Annotated ID	Gene	Base Change^1^	Amino Acid Change
*lpg0324*	*rpsL*	A 378,931 G	K 88 R
*lpg0568-lpg0569*	intergenic	AGAT 608,162–608,165 GAAG	None.
*lpg0671*	*ndh*	721,345–721,353 del	AEI 453 del
*lpg0716*	hypothetical protein	C 782,280 T	P 283 S
*lpg0718*	proton/sodium glutamate symport protein	C 783,398 T	Synonymous
*lpg1228-lpg1271*	*lvh* region	1,355,533–1,401,010 del	Not applicable
*lpg2506*	*luxN*	C 2,825,337 A	A 99 D
*lpg2669*	*ftsE*	T 3,016,381 C	T 206 A

1 Nucleotide positions within the published *L. pneumophila* Philadelphia-1 genome [18] (GenBank accession AE017354.1).

### Parallel Mutations in Two Independent Domestications of Philadelphia-1

Despite being independently derived in two separate laboratories, both JR32 and Lp01 contain the same mutation K88R in *rpsL*, the 30S ribosomal protein. This substitution has been frequently observed in streptomycin resistant bacteria [22]–[24], consistent with streptomycin selection being the first step in the generation of both JR32 and Lp01 (**fig. 1A** **, step 1**). To directly test this hypothesis, on three separate occasions we derived a total of 40 spontaneous streptomycin resistant clones from the Philadelphia-1 progenitor strain by plating onto streptomycin containing solid medium. We next PCR amplified and sequenced the *rpsL* locus in each of these strains. Isolates from the same experiment that contained identical mutations were only counted once in our analysis, as repeated isolation of the same sequence in one experiment could result from overrepresentation of a single streptomycin resistant founder clone. Of the seven clones guaranteed to be independently derived, 3 clones carried the same K88R mutation as JR32 and Lp01. Of the remaining four clones, two carried a K43R mutation, one carried a K43N mutation, and one carried a K43T mutation. As expected, none of the streptomycin resistant clones examined were wild-type at the *rpsL* locus.

**Figure 2:**
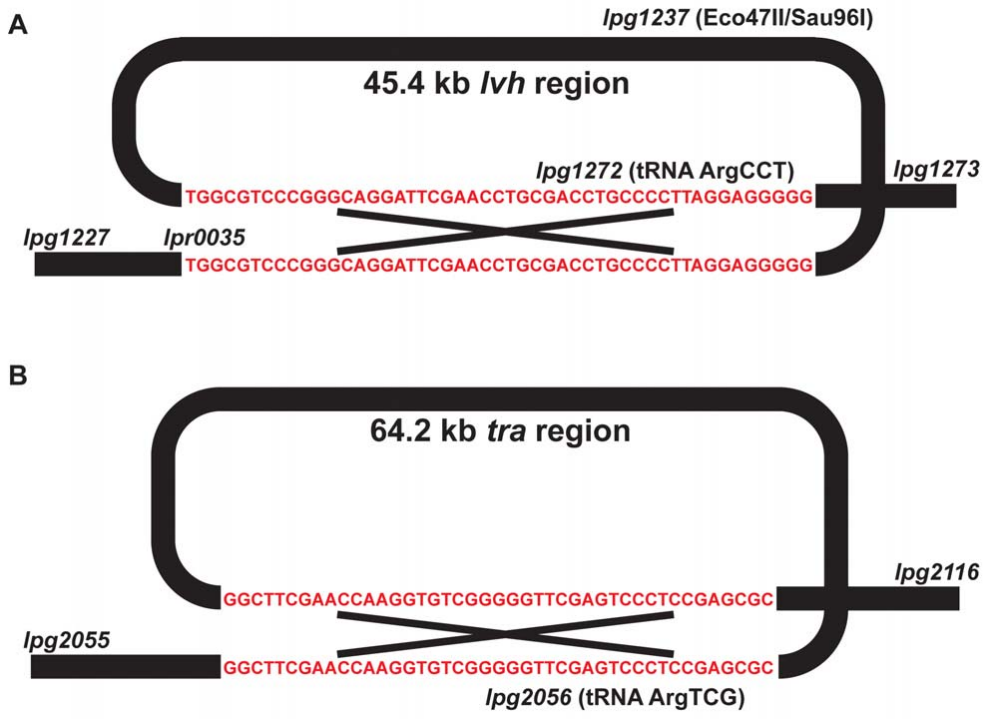
Short, GC-rich direct repeats flank each of the large genomic regions lost in the Lp01 and JR32 lineages. (A) The 45.4 kb *lvh* region that is deleted in the Lp01/Lp02/Lp03 lineage is flanked by a set of 69% GC, 49 nt direct repeats that likely facilitated intrachromosomal recombination and loss of the *lvh* region, which includes a type IVA secretion system as well as a restriction endonuclease, *lpg1237*, and its cognate methytransferase, *lpg1236*. (**B**) The 64.2 kb *tra* region is flanked by a set of 67% GC, 43 nt direct repeats that share no sequence homology with the *lvh* repeat. The genomic sequence of JR32 is consistent with an intrachromosomal recombination event between these repeats and subsequent loss of the intervening *tra* region.

Relative to their clinical ancestor, both JR32 and Lp01 display improved transformation efficiencies using exogenous DNA [8], [9], a presumptive consequence of spontaneous restriction-minus mutations that were selected for during the differentiation of each strain (**fig. 1A** **, step 2**). Consistent with these observations, the JR32 genome contains a mutation in *lpg1237*, a predicted Eco47II/Sau96I restriction endonuclease. While the increased transformation efficiency of this strain would be phenotypically consistent with a loss-of-function mutation in this restriction enzyme, the JR32 polymorphism in *lpg1237* is a nonsynonymous G90E point mutation, suggesting an indispensible function for this particular residue. Lp01 does not carry a specific mutation in this restriction enzyme, but instead carries a 45.4 kb deletion that removes the gene, along with *lpg1228* to *lpg1271*, inclusively (**fig. 2A**). Notably, this region also contains several genes with homology to the Vir type IV translocation system [25]. In addition to removing *lpg1237*, the restriction endonuclease, this large deletion also removes its cognate DNA methyltransferase, *lpg1238*. As was previously described [26], [27] this region is flanked by a high GC% 49 nt direct repeat in the Philadelphia-1 progenitor – a genomic feature within a duplicated arginine tRNA that is thought to support a transition to episomal maintenance of this fragment [26], [27] and likely facilitated the loss of this entire region during the selection for maintenance of exogenous plasmid DNA in the Lp01 lineage (**fig. 1A** **, step 2**). PCR amplification using primers flanking this region, followed by Sanger sequencing, was used to confirm the loss of one of the two direct repeats along with all of the intervening nucleotides corresponding to positions 1355582–1401010 of the Philadelphia-1 reference genome.

We examined the JR32 genome for similar stretches of low coverage and identified a 64.2 kb region deletion spanning *lpg2056* to *lpg2115* (**table 2**). This region contains several *tra* genes with homology to the F-plasmid transfer region. The inability to detect these genes in the JR32 strain had several years ago led some to suggest that this laboratory strain might not actually have been derived from Philadelphia-1 [28]. Like the *lvh* region, this region contains a cluster of proteins with homology to a type IV translocation system. Whole-genome alignments confirmed that the *tra* region is present in both the Philadelphia-1 ancestral strain and Lp01.

We next asked if specific flanking sequences are present in the Philadelphia-1 ancestral strain that could have facilitated the loss of the *tra* region during the domestication of JR32. Similar to the *lvh* region in Lp01, this locus is flanked by a 43 nt set of high GC% direct repeats in the Philadelphia-1 ancestor at an arginine tRNA (**fig. 2B**). We hypothesized that the JR32 *tra* deletion, like *lvh* in Lp01, might occur from an intragenic recombination between these repeats. To confirm the exact location of the deletion in this region, we PCR amplified JR32 template DNA using primers flanking each of these 43 nt repeats. Sanger sequencing of this product confirmed the loss of one of the two direct repeats, along with all of the intervening nucleotides corresponding to positions 2296869–2361031 of the clinical reference genome. Notably, the 43 nt direct repeats flanking the *tra* region share no significant sequence homology to the 49 nt repeats that flank the *lvh* region in the Philadelphia-1 ancestor.

### Comparative Genomic Analysis of Two Widely-used Lp01 Derivatives, Lp02 and Lp03

We next used the same approach to sequence and analyze two commonly-used derivatives of Lp01, the laboratory strains Lp02 and Lp03 [9]. Lp02 is a thymidine auxotroph generated by plating Lp01 onto trimethoprim and thymidine solid growth media [9] (to select for spontaneous thymidine auxotrophs, which are resistant to trimethoprim treatment [29]) (**fig. 1A** **, step 3**). Lp03 is a Dot/Icm translocation deficient mutant that since its isolation has been widely used as an avirulent and/or translocation deficient control [30]–[33]. Lp03 is a spontaneous *dotA* mutant that was isolated based on its resistance to thymineless death in U937 macrophages (**fig. 1A** **, step 4**) [9]. Remarkably, Lp03 was isolated as part of a transposon mutagenesis screen in which it was believed that the Tn5 transposon failed to integrate and mobilize [9]. Our data confirm the absence of any Tn5 sequence in Lp03, as 0 out of 8.8 million reads mapped to Tn5 (GenBank accession U00004.1). Mutations in the Lp02 and Lp03 genomes relative to the Philadelphia-1 progenitor are listed in **table 4** and **table 5**, respectively.

**Table 4:** Lp02 polymorphisms relative to the Philadelphia-1 progenitor.

Annotated ID	Gene	Base Change^1^	Amino Acid Change
*lpg0324*	*rpsL*	A 378,931 G	K 88 R
*lpg0671*	*ndh*	721,345–721,353 del	AEI 453 del
*lpg1228-lpg1271*	*lvh* region	1,355,533–1,401,010 del	Not applicable
*lpg1348*	*leuS*	A 1,487,960 G	V 53 A
*lpg2783*	*nuoG*	T 3,135,908 C	R 259 G
*lpg2868*	*thyA*	G 3,246,196 A	Q 33 ochre

1 Nucleotide positions within the published *L. pneumophila* Philadelphia-1 genome [18] (GenBank accession AE017354.1).

**Table 5:** Lp03 polymorphisms relative to the Philadelphia-1 progenitor.

Annotated ID	Gene	Base Change^1^	Amino Acid Change
*lpg0324*	*rpsL*	A 378,931 G	K 88 R
*lpg0671*	*ndh*	721,345–721,353 del	AEI 453 del
*lpg1228-lpg1271*	*lvh* region	1,355,533–1,401,010 del	Not applicable
*lpg2686*	*dotA*	G 3,037, 018 A	Q 188 ochre
*lpg2783*	*nuoG*	T 3,135,908 C	R 259 G
*lpg2866*	*thyA*	3,245,790–3,245,791 del	Frameshift after residue 167.

1 Nucleotide positions within the published *L. pneumophila* Philadelphia-1 genome [18] (GenBank accession AE017354.1).

### Genomic Inconsistencies with the Reported Histories of Several Laboratory Strains

**Figure 3:**
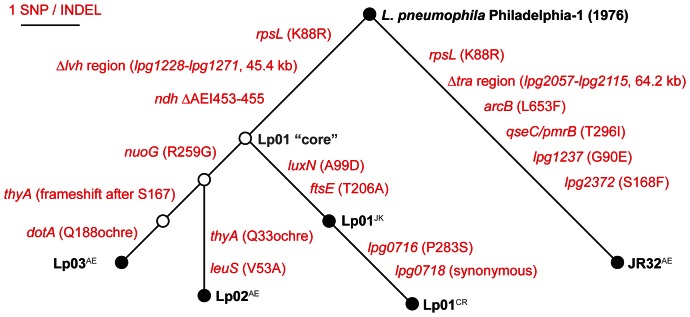
Phylogeny of the *Legionella pneumophila* Philadelphia-1 laboratory strains as determined by whole-genome Illumina sequencing. Illumina sequencing was performed on the Philadelphia-1 progenitor and each of its laboratory derivatives, JR32, Lp01, Lp02, and Lp03. Each genome was aligned to the published *L. pneumophila* Philadelphia-1 sequence (GenBank accession AE017354.1). The identification of single nucleotide polymorphisms (SNPs) and insertions and deletions (INDELs) in each laboratory strain was used to determine genetic distances with maximum likelihood between each strain. To explain these relationships, intermediate ancestral strains were proposed, as represented by open circles. A previously sequenced Lp01 derivative (Lp01^JK^) containing *luxN* and *ftsE* mutations, but not *lpg0716* and *lpg0718* mutations indicates that *luxN* and *ftsE* emerged first within the Lp01 lineage [7]. Not displayed: a putative gene conversion event that emerged independently in only the Lp01^CR^ and JR32 strains (AGAT 608,162–608,165 GAAG). (For details of each exact isolate sequenced, see materials and methods: JR32^AE^, Lp01^CR^, Lp01^JK^, Lp02^AE^, Lp03^AE^).

The published descriptions of Lp01, Lp02, and Lp03 predict a simple phylogenetic relationship as outlined in **fig. 1A**. We used our comparative genomic data to reconstruct the relationship between each of these strains and observed several notable differences between the predicted phylogeny and this reconstruction (**fig. 3**). For instance, the Lp01 genome that we sequenced contains 4 mutations that are not present in either Lp02 or Lp03, consistent with the acquisition of each of these mutations after the derivation of Lp02 from Lp01. Two of these mutations, *lpg0716* and *lpg0718* were identified in our Lp01 strain (Lp01^CR^) but not in a different Lp01 strain from the Isberg lab collection that underwent less laboratory passage (Lp01^JK^) [7]. This indicates that the mutations in *lpg0716* and *lpg0718* were acquired after mutations in both *luxN* and *ftsE* in the Lp01 lineage. Similarly, Lp02 and Lp03 share a *nuoG* mutation, unlinked to thymidine auxotrophy, that separates these lineages from their last common ancestor. Like Lp01, the strain of Lp02 that we sequenced appears to have acquired an additional mutation since the derivation of Lp03 from this *nuoG* ancestor: a mutation in *leuS*. Based on comparative data between Lp01 and its derivatives, Lp02 and Lp03, we can define the “core” Lp01 genome as having mutations in *rpsL* and *ndh*, along with the large *lvh* deletion relative to the Philadelphia-1 ancestor (**fig. 3**).

Both Lp01 and JR32 also contain an AGAT to GAAG substitution upstream of *lpg0569*, one of three copies of the 16S rRNA gene. Strikingly, this substitution is not present in any of the other strains that we have sequenced (Philadelphia-1, Lp02^AE^, Lp03^AE^, and Lp01^JK^) [7] (**fig. 3**). Consistent with the published sequence, the same GAAG allele is upstream of the other 16S rRNA loci, suggesting that the Lp01 and JR32 substitutions may have occurred due to intragenic gene conversion [34].

Both Lp02 and Lp03 are thymidine auxotrophs due to selection for spontaneous *thyA* mutations on trimethoprim [9] (**fig. 1A** **, step 3**). Lp03 is reported to be a spontaneous *dotA* mutant derived from Lp02 [9]. Notably, our sequencing indicates that while Lp02 and Lp03 both contain mutations in *thyA* predicted to inactivate the protein, these mutations are genetically distinct from one another. The *thyA* mutation in Lp02 results in a premature stop codon at residue 33, whereas the *thyA* mutation in Lp03 results in a (−2) frameshift after residue 167. These results are inconsistent with the derivation of Lp03 from Lp02, but rather suggest parallel selection of two independent *thyA* mutants on trimethoprim from the same *nuoG* progenitor.

### The *L. pneumophila* Philadelphia-1 Progenitor Tolerates Transformation

Due to the significant differences between the progenitor Philadelphia-1 isolate and both Lp01 and JR32, we next asked whether the non-domesticated strain might be an appropriate laboratory substitute in some circumstances. One reasonable argument for using a restriction-minus domesticated strain would be that introduction of foreign DNA is presumably well tolerated. Conversely, introduction of foreign DNA into the non-domesticated Philadelphia-1 progenitor might lead to unpredictable consequences, making direct comparison between manipulated strains difficult. To test whether this might be the case, we used electroporation to introduce a 8.9 kb GFP-expression plasmid into the undomesticated Philadelphia-1 strain and subjected 14 chloramphenicol resistant, GFP-positive transformants to whole-genome resequencing. Based on our analysis of the Lp01 and JR32 lineages, we expected to identify restriction-minus mutations in most of the strains, but reasoned that by sequencing several transformants, we could determine the relative likelihood and likely effects of each mutation.

Illumina reads from each of the transformed Philadelphia-1 strains were reference assembled to the published *L. pneumophila* str. Philadelphia-1 genome. Remarkably, with the exception of 1 out of the 14 strains, all of the transformed strains are completely identical at the nucleotide level, suggesting that the introduction of this plasmid by electroporation and its subsequent maintenance is well tolerated by these strains. (The one mutation in this transformed strain was a T95A substitution in a phosphomannomutase, *lpg2486*.) While it was previously suggested that introduction of plasmid DNA by electroporation might lead to secondary mutations in the recipient strain [35], we take our data to suggest that the acquisition of secondary mutations is not a general feature of *L. pneumophila* electroporation. These results stand in stark contrast to the observation that the conjugative introduction of pMR5, a 60 kb plasmid, resulted in restriction-minus mutations in the Lp01 and JR32 lineages [8], [9]. Perhaps pMR5 selected for these mutations in Lp01 and JR32 due to the proportionally increased number of internal restriction sites harbored on such a large plasmid.

We next used *de novo* assembly (see Materials and Methods) to generate the circular plasmid sequence from the set of reads that did not align to the Philadelphia-1 genome. Reference assembly to this circularized, assembled plasmid sequence confirmed non-mutated plasmid sequence in each of the 14 clones. We used average depth-of-coverage to estimate plasmid copy number in each of these transformed clones, with the ratio of plasmid to genome coverage ranging from 2.9 to 18.8-fold (mean 7.6).

## Discussion

The present accessibility and affordability of whole-genome sequencing provides a unique opportunity to ensure the genomic integrity of strains during genetic manipulation in the laboratory and incidental passage. Researchers should consider at what point routine genomic sequencing of strains should become part of their general workflow to supplement or replace traditional microbiology approaches, such as comparing results from multiple, independently-derived clones. Some laboratories have already begun to sequence individual knockout strains to make sure that they do not harbor background mutations that might otherwise complicate their analysis [36], [37].

While both Lp01 and JR32 are derived from clinical strains isolated during the 1976 outbreak [8], [9], the exact relationship between the two progenitors is unknown. JR32 was derived in the Shuman laboratory from a 1976 clinical isolate provided by Marcus Horwitz, who himself had earlier received the strain from the Centers for Disease Control [38]. Lp01 is derived from an isolate independently provided to Ralph Isberg directly by the CDC [9]. The previously published Philadelphia-1 genome is based on the Horwitz clinical strain [18], allowing us to ask the question, “Were the strains given to each laboratory the same?” Notably, each of the polymorphisms that we identified between the Isberg laboratory's Philadelphia-1 strain and the published (Horwitz) Philadelphia-1 genome are also present in the JR32 strain (**tables 1** **–** **3**). Thus, despite uncertainties concerning the relative provenance of each progenitor strain, our results indicate that these two laboratory lineages were likely derived from genetically identical clinical isolates.

Our resequencing of the *L. pneumophila* Philadelphia-1 genome using the Illumina platform identified several minor polymorphisms between the published sequence [18] and our draft consensus. We suspect that most, if not all, of the polymorphisms identified in **table 1** are sequencing errors refined by this new effort. (The alternative explanation for such discrepancies is the emergence of mutations in the Horwitz Philadelphia-1 isolate after the derivation of JR32 [8] but before the sequencing of the strain by Chien *et al* [18]). These differences ranged from the trivial (the “identification” of the last ambiguous nucleotide in the genome, N 1,355,972 T) to the functionally significant (the fusion of *lpg0644* and *lpg0645* to form a complete *rtxA* open reading frame). These preliminary results suggest that the time has come for a systematic reannotation of the Philadelphia-1 genome. Since its initial description, several other *L. pneumophila* genomes have been finished that would support the identification of misannotated loci through comparative genomic analysis. Indeed, in our non-exhaustive analysis of intergenic polymorphisms, we have already identified intact open reading frames corresponding to an unannotated Philadelphia-1 ortholog of the *fur* gene (between *lpg0372* and *lpg0373*) [7] and another between *lpg0745* and *lpg0746* with 100% protein homology to the *lpp0811* locus from *L. pneumophila* Paris (this study).

Whole-genome resequencing has emerged as a powerful tool for tracking bacterial transmission and evolution during outbreaks of disease [14]–[17], [39], [40]. We have taken a similar approach to trace the evolutionary trajectory of *L. pneumophila* Philadelphia-1 during its domestication. In particular, by analyzing several strains derived from the same laboratory lineage (Lp01, Lp02, and Lp03), we identified the last common ancestor of all three strains, despite the physical absence of such a strain from our collection (**fig. 3**). This “core” Lp01 genome contains only three mutations that separate it from its Philadelphia-1 progenitor: an *rpsL* point mutation linked to streptomycin resistance, a 45.4 kb deletion that elements both a restriction system and an accessory type IV secretion system, and an in-frame 3 amino acid deletion in the NADH dehydrogenase transmembrane protein, *ndh*. Based on the shared ancestry of this “core” Lp01 strain and its derivatives, Lp02 and Lp03, we anticipate that every Lp01 strain around the world will share, at a minimum, this common set of three mutations. Two additional mutations in the contemporary Lp01 strain, substitutions in *luxN* and *ftsE*, were also observed in a distinct Lp01 strain that we used in our experimental evolution of *L. pneumophila* to mouse macrophages [7], presumably indicating that this particular clone has undergone less incidental laboratory passage.

One of the surprising results from our phylogenetic reconstruction of the Lp01, Lp02, and Lp03 lineages is the relationship between Lp02 and Lp03. The last common ancestor between Lp02 and Lp01 is the “core” Lp01 strain, reflecting the fact that this strain was presumably derived from Lp01 prior to the incidental laboratory passage that led to the acquisition of the 4 additional mutations present in the contemporary Lp01 strain. Lp03 is a spontaneous *dotA* mutant reportedly derived from Lp02 [9], yet our analysis strongly suggests that this is not the case. Lp02 and Lp03 are both thymidine auxotrophs, yet genomic sequencing indicates that they each contain a different mutation in the *thyA* locus. The simplest explanation for this event is that Lp03 was not actually derived from Lp02, but rather from a different trimethoprim resistant clone selected at the same time.

Most of the polymorphisms between the domesticated lineages are small and thus would only be detected by the resolution provided by whole-genome resequencing, however two large genetic lesions were also observed. In the Lp01 lineage, a 45.4 kb deletion spanning both the restriction system and several genes with homology to the Vir Type IV secretion system (*Legionella vir* homologs, *lvh*) [25] was detected. The formation of this lesion, which has been previously described [25], [27], is likely facilitated by a set of GC-rich direct repeats that flank the region in the Philadelphia-1 progenitor and are believed to facilitate the transition of the *lvh* region from an integrated to episomal state [12], [26], [27], [41]–[43]. The loss of this region in the Lp01 lineage has been hypothesized to be a consequence of the selection for a spontaneous restriction-minus clone [12] (**fig. 1A** **, step 2**) and the reconstructed phylogeny of this lineage supports this hypothesis. It is less clear if this region had to go through an episomal intermediate as part of its loss or if a direct intrachromosomal recombination event led to the deletion.

Not to be outdone, the JR32 lineage contains a large, 64.2 kb deletion relative to the Philadelphia-1 progenitor strain, covering a different type IV secretion system encoded for by the *tra* genes. While the absence of the *tra* region in the JR32 strain was previously described, lack of genomic data at the time led to some confusion concerning the relationship between JR32 and *L. pneumophila* Philadelphia-1, with some suggesting a different ancestry for JR32 [28]. Our data support the model that, like the *lvh* region in Lp01, the loss of this region was facilitated by a set of flanking GC-rich repeats (**fig. 2**). While the *tra* region is known to exist in an episomal state in *L. pneumophila* Corby [44], [45], it is unclear whether this region retains a replication origin and the ability to exist in an episomal state in the Philadelphia-1 genetic background. Furthermore, unlike the *lvh* region, the selective pressures that led to the loss of the *tra* genes are less clear. While JR32 contains a point mutation in the *lpg1237* restriction endonuclease that is presumably responsible for its restriction-minus phenotype, we hypothesize that the *tra* region was lost during JR32 domestication due to indirect effects either from plasmid introduction, maintenance, or curing at 40°C. Notably, a survey of environmental and clinical isolates of *L. pneumophila* previously observed that sequences contained within the *tra* region were less associated with clinical than environmental strains [42].

As expected, sequencing the domesticated strains of *L. pneumophila* Philadelphia-1 also identified several mutations that do not appear to be linked to specific prescribed events (**fig. 1A**), but instead may have emerged as a result of inadvertent laboratory passage (**fig. 3**). Some of these mutations, such as a synonymous mutation in *lpg0718*, might be the result of random genetic drift, though others might reflect adaptations to the specific requirements of *in vitro* growth. *L. pneumophila* is thought to be severely limited in its ability to replicate extracellularly in the environment [4], making the *in vitro* culture of these bacteria even more of a novel growth environment than what might be expected for less fastidious organisms. Non-synonymous mutations in 8 genes were identified in the domestic lineages that have no clear connection to a selective event: *arcB*, *ftsE*, *luxN*, *ndh*, *nuoG*, *qseC* (*pmrB*), *lpg0716*, and *lpg2372*. Similar to what we have observed with experimentally evolved *L. pneumophila* [7], none of these mutations is an obvious loss-of-function mutation. Re-examination of published transposon mutagenesis data [36] indicates that insertions in only one of these genes, *luxN*, led to improved extracellular growth. In contrast, insertions in two of the genes, the NADH dehydrogenase subunit, *nuoG*, and the two-component quorum sensing kinase, *qseC*, resulted in severe extracellular growth defects [36]. Because laboratory propagation of *L. pneumophila* occurs under the same conditions used during the generation of this transposon library, these results argue that the Lp01 *luxN* mutation may approximate the null phenotype of this gene, whereas neither the *nuoG* (Lp02) or *qseC* (JR32) mutations are null alleles. These results are consistent with our earlier data that suggested that the functional consequences of spontaneous, adaptive mutations infrequently reflect null, complete loss-of-function phenotypes [7].

Among the spontaneous mutations in both the JR32 and Lp01 lineages, some common themes can be observed. In the Lp02 lineage, the sequential acquisition of mutations in the NADH dehydrogenase subunits *ndh* and *nuoG* might reflect a change in oxygen tension for these bacteria cultured under laboratory conditions [41]. Consistent with this hypothesis, we also identified a mutation in the aerobic respiration control sensor kinase, *arcB* [46], [47], in the JR32 lineage. In both the JR32 and Lp01 lineages, additional mutations in response regulators were seen, each of which could be predicted to have a significant impact on the response of these strains to different environments. In the Lp01 lineage, the sensor histidine kinase/response regulator *luxN* is mutated, whereas in JR32, in addition to *arcB*, we observe mutations in the global regulator *qseC* (*pmrB*) [48].

A number of phenotypic differences have been described between Lp01, Lp02, JR32, and their Philadelphia-1 progenitor. It has been reported that Lp01 displays reduced adherence to host cells, reduced intracellular replication in U937 macrophages and *Acanthamoeba castellanii*, reduced replication in the murine lung, and increased rates of lysosomal fusion [12]. When each strain was grown in broth to control for bacterial growth phase, a known determinant of *L. pneumophila* virulence [49], at least one of these differences (adherence) disappeared [12]. Others have reported several orders of magnitude increased natural transformation efficiency for Lp02 relative to both JR32 and the Philadelphia-1 progenitor [50]. In another striking difference, the Dot/Icm component, *dotL*, was shown to be essential for viability in the Lp02 strain background, but not in JR32 [51]. The availability of whole-genome sequence for each of the Philadelphia-1 domesticated strains should assist in linking each of these phenotypes to specific genetic differences.

The genetic distance between both Lp01 and JR32 and the *L. pneumophila* Philadelphia-1 progenitor raises the obvious question, “Is domestication of *L. pneumophila* necessary?” Using whole-genome resequencing, we observed that the electroporation of an 8.9 kb plasmid into the restriction-positive Philadelphia-1 strain was well tolerated and rarely led to the acquisition of secondary mutations in either the plasmid or the genomic sequence. This suggests that it may be worth revisiting the general applicability of the Philadelphia-1 progenitor for genetic manipulation and laboratory study. Through either natural transformation [35] or electroporation of a suicide plasmid it should be possible to directly construct an *rpsL*, *lpg1237* knockout strain of *L. pneumophila* Philadelphia-1 that retains the high transformation efficiencies of Lp01 and JR32 yet is minimally separated from the wild-type clinical background. For most applications, such manipulation may not even be necessary. Indeed, a related strain, *L. pneumophila* Paris, has been routinely genetically manipulated without any engineering to make it restriction-minus [52], [53]. One potential risk of this approach might be unpredictable genetic instability, yet our whole-genome sequencing of 14 electroporated clones indicated that the introduction of an 8.9 kb plasmid did not lead to spontaneous, unpredictable mutations. In further support of working directly with undomesticated strains, others have reported efficient electroporation of a different 10 kb plasmid into a Philadelphia-1 clinical strain [54]; we were also able to use natural transformation to introduce a gentamicin resistant marker into the locus of *dotA* in our Philadelphia-1 clinical strain (data not shown).

For those seeking to utilize clinical Philadelphia-1 in their studies, it is important to note that the only publically available Philadelphia-1 strain (ATCC 33152) is not a genetic match to the *L. pneumophila* Philadelphia-1 strains used to derive the JR32 and Lp01 laboratory strains. Alignment of the *dotA* gene sequence from ATCC 33152 (GenBank AF095231.1) [55] to the published Philadelphia-1 sequence (GenBank AE017354.1) demonstrates that these two strains are indeed distinct. While both of these strains are from the 1976 outbreak [18], [55], genomic data from other outbreaks of bacterial disease has clearly shown that not all isolates from the same outbreak are genetically identical [14]–[16], [40].

## Materials and Methods

### Bacterial Strains

*Legionella pneumophila* Philadelphia-1 is a previously described isolate from the 1976 outbreak of Legionnaires’ disease in Philadelphia, Pennsylvania USA and was a kind gift from B. Fields (CDC) to Ralph Isberg. Lp01, Lp02, and Lp03 are laboratory derivatives of Philadelphia-1, all derived in the Isberg laboratory as previously described [9]. Lp01^CR^ is the Lp01 strain in Chitong Rao's strain collection, given to him from Alexander Ensminger. Lp01^JK^ is an Lp01 isolate, JK240, frozen by James Kirby during his time as an Isberg postdoctoral researcher. Lp02^AE^, Lp03^AE^, and JR32^AE^ are from Alexander Ensminger's strain collection, frozen during his postdoctoral training in the Isberg laboratory. JR32 was a kind gift from H. Shuman to the Isberg laboratory and was isolated as previously described [8].

### Plasmids

pPpacS-EGFP is a 8946 bp non-integrating plasmid, generated by replacing the IPTG-inducible promoter in a *mobA* version of the pAM239 plasmid [56] with the native *Legionella* promoter upstream of the highly expressed gene, *pacS*. The PpacS promoter was cloned from a mouse macrophage-adapted strain of *L. pneumophila* [7] and contained a single nucleic acid change relative to the wild type corresponding to a 1 nt deletion at position 3,041,723 in GenBank AE017354.1 that increased the transcription of *pacS* by over 5-fold (data not shown). Forward primer 5′-CAGGGCCCAATGTGTTCTGCTCCAAAAATTA-3′ and reverse primer 5′-GCTCTAGAAATCGCTCGCTGAGTTAAATG-3′ were used to PCR amplify the 358 nt fragment from 100 ng of genomic DNA with KAPA HiFi HotStart ReadyMix (Kapa Biosystems). The PCR product of the PpacS promoter and the plasmid vector pAM239 were digested with ApaI and XbaI, and then ligated and transformed into TOP10 *E. coli* by heat shock. Selection of transformants on LB plates was performed with 34 µg/ml chloramphenicol. pPpacS-EGFP plasmid was midiprepped from these cells using the GenElute Midiprep kit (Sigma) according to the manufacturer's suggestions. *De novo* assembly of paired-end Illumina reads from pPpacS-EGFP transformed *L. pneumophila* strains (see below) was used to determine the complete sequence of this plasmid.

### Electroporation

An overnight culture of *L. pneumophila* Philadelphia-1 was grown overnight from a single 2 day old patch in ACES-buffered yeast extract (AYE) liquid growth medium [57] at 37°C under atmospheric conditions to exponential phase (A600 nm ∼ 2.0). 1 ml of culture was centrifuged at 12,000×g for 1 minute at 4°C. Pellets of bacteria were washed with 1 ml of ice-cold sterile ultrapure water, centrifuged as before, and then rinsed again (2 washes with 1 ml ice-cold water each). After an additional wash in 1 ml of ice-cold, 10% glycerol, each pellet was resuspended in 100 µl of ice cold, 10% glycerol. 50 µl of each resuspension was mixed gently with 100–200 ng of plasmid DNA and transferred to an ice-cold, 2 mm gap electroporation cuvette (VWR). Electroporation was performed on a ECM 630 (BTX) electroporator set to 600 ohms, 25 µF, 2.5 kV and cell pellets were streaked to single colony onto charcoal buffered ACES yeast extract (CYE) plates [58] containing 5 µg/ml chloramphenicol and incubated at 37°C for 4–5 days.

### Illumina Genomic Library Preparation and Sequencing

Custom Illumina libraries for Philadelphia-1, JR32-AE, Lp01-CR, Lp02-AE, Lp03-AE, each of the 14 pPpacS-GFP plasmid transformed clones, and two untransformed controls were generated largely as previously described [7]. Briefly, genomic DNA was prepared from post-exponential (A600 nm >4) bacteria using the Qiagen DNeasy kit including the optional RNase digestion step (Ambion RNase cocktail) and eluting in ultrapure water (Life Technologies). Genomic DNA was quantified on a Nanodrop 2000C UV-Vis Spectrophotometer (Thermo Scientific) and either concentrated by speed vac or diluted to a final concentration of 40 ng/µl in a total volume of 130 µl of ultrapure water. DNA was sheared using a Covaris S2 ultrasonicator according to the manufacturer's directions for 400 bp DNA fragmentation. Sheared DNA was concentrated by speed vac to 30 µl total volume, then treated with the End-IT DNA Repair kit (Epicentre) for in a 50 µl volume for one hour at room temperature. After spin-column purification using the Machery-Nagel NucleoSpin kit according to the manufacturer's directions, DNA was eluted in 30 µl of elution buffer. 3′ A-tailing was performed by incubating for 1 hour at room temperature with Exo-minus Klenow (Lucigen) and dATP. Samples were purified using the Qiagen MinElute kit and eluted in a total of 10 µl of elution buffer. Custom adapter sequences with 8 nucleotide indices located 3′ of each insert were ligated to each sample using the Fast-link ligation kit (Epicentre) with overnight 16°C incubations. Ligated DNA of approximately 500–600 nt of total length was isolated by 2% agarose TAE gel electrophoresis, with narrow bands excised using GeneCatcher disposable gel excision tips (the Gel Company) and purified using the Machery Nagel NucleoSpin kit. To enrich for properly ligated samples, approximately 4% of each library was amplified for 16 cycles using common primers, purified using the NucleoSpin kit, and then quantified using a Nanodrop spectrophotometer. Libraries were diluted to 20 nM and mixed in equimolar amounts prior to sequencing. The Philadelphia-1 progenitor and Lp01 strains were sequenced as part of a multiplexed MiSeq (Illumina) 250×8×250 run. All the other strains were sequenced on a partial lane of a multiplexed HiSeq 2500 (Illumina) 100×8×100 run. All sequencing was performed at the Donnelly Sequencing Centre at the University of Toronto.

### Assembly Software and Parameters

Raw paired-end reads from the MiSeq or HiSeq runs were reference assembled using Geneious R6 (Biomatters Ltd) to the published *L. pneumophila* Philadelphia-1 genome (GenBank accession AE017354.1) under “medium sensitivity” which corresponds to the following parameters: maximum gaps per read (15%), maximum gap size (50), minimum overlap (25), minimum overlap identity (80%), word length (14), index word length (12), ignore words repeated more than (10 times), maximum mismatches per read (30%), maximum ambiguity (4), with paired-reads, and multiple best matches excluded from the assembly. Mean genomic depth of coverage ranged from 101 to 302 in all the libraries. Unassembled reads from one of the of the pPpacS-GFP transformed genomes were *de novo* assembled using Velvet [59] into a single contig. This contig was used to generate a circular map of the plasmid, with part of the *L. pneumophila pacS* promoter sequence (that would not end up in the unassembled reads) manually added to close the gap at each end and circularize the sequence. Reads from each GFP-transformed clone were also aligned to this sequence to confirm stable maintenance of intact plasmid DNA. Lp03 reads were also reference assembled to Tn5 (GenBank accession U00004.1) to confirm that no Tn5 sequence was present in that strain. Genetic variation discovery was performed at frequencies of 0.8 with coverage no less than 30% of the mean depth for the genome. Within Geneious, the position of each polymorphism identified in one strain was manually visualized in all other strains to confirm the presence or absence of each polymorphism across strains.

### Literature Survey for Lp01/Lp02 and JR32 Lineages

A Google Scholar search was performed (on February 24, 2013) for the terms “Legionella JR32 -Lp01 -Lp01”; “Legionella Lp01 OR Lp02 -JR32”; and “Legionella JR32 Lp01 OR Lp02.” 239 citations mentioned Lp01 or Lp02 without JR32. 409 citations mentioned JR32 without Lp01 or Lp02. 53 citations mentioned JR32 and at least one of Lp01 or Lp02.

### Data Accessibility

GenBank (.gbk) formatted assemblies of the genomes of each laboratory strain have been deposited into the Dryad Digital Repository (doi:10.5061/dryad.1k1ns). These files, generated by editing the published Philadelphia-1 genome (GenBank AE017354.1) in Artemis [60], can be used for visualization or resequencing experiments by opening them with any of a number of commercial or freely-available genome browsers and assembly programs - such as Artemis [60], CLC Sequence Viewer (CLCbio), CLC Genomics Workbench (CLCbio), or Geneious (Biomatters Ltd). The 21 raw paired-end sequence reads used in this study were all deposited as Illumina FASTQ files to the NCBI sequence read archive (Study accession: SRP020472).
